# Where Taxonomy Based on Subtle Morphological Differences Is Perfectly Mirrored by Huge Genetic Distances: DNA Barcoding in Protura (Hexapoda)

**DOI:** 10.1371/journal.pone.0090653

**Published:** 2014-03-07

**Authors:** Monika Carol Resch, Julia Shrubovych, Daniela Bartel, Nikolaus U. Szucsich, Gerald Timelthaler, Yun Bu, Manfred Walzl, Günther Pass

**Affiliations:** 1 Department of Integrative Zoology, University of Vienna, Vienna, Austria; 2 Institute of Systematics and Evolution of Animals, Polish Academy of Sciences, Krakow, Poland; 3 Institute of Plant Physiology & Ecology, Shanghai Institutes for Biological Sciences, Chinese Academy of Sciences, Shanghai, People's Republic of China; Roehampton university, United Kingdom

## Abstract

**Background:**

Protura is a group of tiny, primarily wingless hexapods living in soil habitats. Presently about 800 valid species are known. Diagnostic characters are very inconspicuous and difficult to recognize. Therefore taxonomic work constitutes an extraordinary challenge which requires special skills and experience. Aim of the present pilot project was to examine if DNA barcoding can be a useful additional approach for delimiting and determining proturan species.

**Methodology and Principal Findings:**

The study was performed on 103 proturan specimens, collected primarily in Austria, with additional samples from China and Japan. The animals were examined with two markers, the DNA barcoding region of the mitochondrial COI gene and a fragment of the nuclear 28S rDNA (Divergent Domain 2 and 3). Due to the minuteness of Protura a modified non-destructive DNA-extraction method was used which enables subsequent species determination. Both markers separated the examined proturans into highly congruent well supported clusters. Species determination was performed without knowledge of the results of the molecular analyses. The investigated specimens comprise a total of 16 species belonging to 8 genera. Remarkably, morphological determination in all species exactly mirrors molecular clusters. The investigation revealed unusually huge genetic COI distances among the investigated proturans, both maximal intraspecific distances (0–21.3%), as well as maximal congeneric interspecifical distances (up to 44.7%).

**Conclusions:**

The study clearly demonstrates that the tricky morphological taxonomy in Protura has a solid biological background and that accurate species delimitation is possible using both markers, COI and 28S rDNA. The fact that both molecular and morphological analyses can be performed on the same individual will be of great importance for the description of new species and offers a valuable new tool for biological and ecological studies, in which proturans have generally remained undetermined at species level.

## Introduction

A great part of the megadiverse arthropods is soil dwelling [1]. The stronger the association to soil the more likely morphological adaptations are to be present, such as elongated or cylindrical body shapes and frequently shortened, reduced or absent body appendages, such as antennae, legs or cerci [2]. In extreme cases this leads to a paucity of morphological characters which impedes species delineation and determination. For many of these taxa only few specialized and experienced taxonomists are able to accurately identify species [3].

In our study we focused on Protura, a poorly known group of primarily wingless hexapods. Due to their small body size (body length between 0.5–2.5 mm) and their hidden life style in the euedaphic soil region our knowledge of their biology and ecology still remains very fragmented (for review see [4]). Proturans are one of the most peculiar groups of hexapods with several unique characters. The most distinguishing character pertains to their front legs, which are held forward and presumably functionally compensate the lack of antenna and its sensory features [5]. The morphology of Protura is very homogeneous and determination is utterly demanding. All important diagnostic characters are difficult to distinguish, such as are the shape of sensilla and their location on the foretarsi and body, the presence or absence of the tracheal system, the structure of abdominal legs, the shape of the maxillary gland, the maxillary and labial palps, the “striate band” on the abdominal segment VIII, the squama genitalis and the porotaxic pattern [6]–[10]. Although Protura is not known from the fossil record, there is general agreement that they represent one of the earliest branches of the hexapod tree, which may date back to 400 million years [11]. Furthermore, their phylogenetic position within hexapods is still a matter of controversy (for review see [4], [12]–[13]).

In the last decades, molecular approaches have attracted attention attempting to facilitate species identification for a broader scientific community including non-experts [14]–[15], (for review see [3]). The most promising and cost-effective approach was proposed by [16]–[17], who introduced the DNA barcoding method, which enables species characterization based on a short DNA region of a universal standardized marker. For animals this marker is represented by a 648 bp long fragment of the mitochondrial cytochrome c oxidase subunit 1 (COI) gene. Additionally, DNA barcoding can support species delimitation of new or cryptic species. Therefore it was suggested to include the COI barcode in any new species description along with knowledge about morphology, geographical distribution, and other ecological and biological data [18].

Since the introduction of DNA barcoding, many studies have ensued and numerous challenges emerged, such as the “barcoding gap”, possible overlaps between intra- and interspecific distances, limitations of a single-gene approach, and alternative or complement markers (for review see [3]).

In the present study, we aimed to establish DNA barcoding for Protura. Until now, the only complete mitochondrial genome was published by [19]. Additionally, few partial sequences are available for cytb, 3′ end of COI, COII and 12S rDNA [19]–[21]. The first complete COI barcodes were published as part of the description of the new species *Acerentulus charrieri* [22], *Yamatentomon guoi* [23], and *Hesperentomon yangi* [24].

Due to the minuteness of proturans, species can only be determined unambiguously after a clearing treatment in which all tissues are removed and subsequent slide mounting of the specimen. Therefore, we adapted a non-destructive DNA-extraction method for Protura to enable the following species determination on basis of the cuticular skeleton [25]. In our barcoding approach we used two molecular markers, the DNA barcoding region of COI and a fragment of the nuclear 28S rDNA, including the Divergent Domains 2 and 3, to investigate whether the species diversity within Protura, as recorded by traditional taxonomy, is reflected in the molecular data.

## Materials and Methods

### Ethics statement

All species used in this study are neither CITES-species nor endangered species according to regional Red List (neither Red List of Austria, nor Red List of the federal states, where the localities lie). As such no special sampling permission is necessary for taking soil samples.

Generally sampling permissions lie in the area of authority of the different federal states of Austria - in our case Vienna, Lower Austria and Carinthia. For Lower Austria our sampling permission number is RU5-BE-939/001-2013.

Soil sampling was performed at the following locations:

### Study sites and sampling

Soil samples were taken from three localities in eastern and southern Austria (i) Leopoldsberg (Vienna, N: 48°16′36.36″ E: 16°21′00.46″) (the worldwide biodiversity hotspot of Protura [26]) collected at 09.09.2009, 13.03.2012, and 23.04.2012, (ii) Eichkogel (Lower Austria, N: 48°03′45.03″ E: 16°17′32.26″) collected at 26.09.2009, and (iii) Twimberger Graben (Carinthia, N: 46°53′54.02″ E: 14°50′54.27″) collected at 24.04.2011 and 01.11.2011. Specimens were expelled from soil samples by Berlese funnels and fixed in 100% EtOH. To enlarge the phylogenetic coverage of the taxon sampling, we additionally included samples from China (*Sinentomon erythranum*) and Japan (*Baculentulus densus*, *Filientomon takanawanum*) (species list Table S1).

### Determination

Species were identified following the determination key for European Protura [7], complemented by details given in [27]–[29].

### DNA extraction, PCR amplification and sequencing

Whole genomic DNA was extracted by a non-destructive extraction method [25]. The procedure follows the standard protocol of the DNA-extraction kit (Blood & Tissue, Qiagen) but with an increased incubation time of 24 hours. The final volume of elution buffer was 60 µl. After DNA-extraction the remaining cuticle was transferred to 96% EtOH, washed in Marc André I, and whole-mounted in Marc André II. In total, 171 individuals were successfully extracted of which 99 were finally sequenced (species list Table S1).

Two thermocycling profiles were used to amplify fragments of COI and 28S rDNA, differing only in the annealing temperature: pre-denaturation at 94°C for 3 min, 35 cycles of 1 min at 94°C, 1 min at 48°C (COI)/ 1 min at 45°C (28S) and 1 min at 72°C, final extension step for 5 min at 72°C. All PCR mixes had a total volume of 25 µl and contained 16.4 µl ddH_2_O, 2 µl template, 2.5 µl primer [10 µM; VbC Biotech], 2.5 µl dNTPs [2 mM each; Fermentas], 2.5 µl PCR Buffer [10x containing 20 mM MgCl_2_; Fermentas DreamTaq], and 1 µl Polymerase [5u/ µl; Fermentas DreamTaq]. In some cases the addition of 0,5 µl MgCl_2_ [25 mM; Fermentas DreamTaq] yielded better results. PCR products were purified using QIAquick PCR Purification kit (Qiagen) and eluted in 35 µl AE buffer. COI and 28S fragments were sequenced in both directions by VbC Biotech Service GmbH (www.vbc-biotech.at/).

A set of different primers was necessary to successfully amplify and sequence fragments of the COI and the 28S rDNA, depending mostly on the genus of the given specimen (Table 1). Regarding the 28S rDNA, the regions 2 and 3 were amplified and sequenced in two overlapping fragments employing three primers specified in [30] and then completed by a new forward primer slightly moved to the 3′ end. In some specimens an additionally designed internal reverse primer was necessary to sequence the whole 28S fragment. COI sequencing reads were assembled and checked by eye for reading frame errors in Bioedit Sequence Alignment Editor [31]. 28S reads were assembled with SeqMan (Lasergene v.8, DNASTAR) and checked by eye.

**Table 1:** List of primer pairs and starting positions within the selected markers used in the present study.

Locus	Primer name	Sequences (5′-3′)	Position (bp)	Reference
COI	LCO1490	ggtcaacaaatcataaagatattgg	23	[29]
	LepF1	attcaaccaatcataaagatattgg	23	[17]
	CamF1	ctcractaaccataargatattgg	26	present study
	ProtF2	acgaaccatagggatatcgg	28	present study
	CamR1	taaacttcdggrtgdccaaaaaatc	707	present study
	ProtR2	gagcycatcatatrtttac	863	present study
	DiplR1	gcaataattatdgtdgctgc	919	present study
28S rDNA	D1a	cccgcgtaatttaagcatat	64	[24]
	D2a	gatagcgaacaagtacc	416	[24]
	D2aprot	gtaccgcgagggaaagttg	428	present study
	D3bint363*	gagcaccgccgaactgtg	545	present study
	D3a	gacccgtcttgaaacacgga	950	[24]
	D3arev*	tccgtgtttcaagacgggac	1092	[24]
	D3arev_prot*	ctccttggtccgtgtttc	1102	present study
	D3b	tccggaaggaaccagctacta	1435	[24]

Reference sequence *Sinentomon erythranum* (NCBI accession number: NC015982), * indicates internal primers additionally used in some specimens.

Sequences from GenBank (NCBI) of the complete mitochondrial genome of *Sinentomon erythranum* (accession number: NC015982, accessible since X.2011) and additionally the COI DNA barcode of *Acerentulus charrieri* (accession number: JQ411217, accessible since III.2012) were downloaded. The sequences of COI determined in this study are deposited at BOLD under the project name PROTAT. The material is deposited in the collection of the University of Vienna and in the collection of the State Museum of Natural History of the National Academy of Sciences of Ukraine, L'viv (SMNH).

### Alignment and data analysis

Alignment and tree reconstruction were performed for each gene separately. The COI sequences were aligned manually in BioEdit Sequence Alignment Editor [31]. The 28S rDNA sequences were aligned using default settings of the program MUSCLE [32], as implemented in Mega v. 5.05 [33]. To avoid errors due to relatively high length variation, subsequently possibly misaligned positions were identified using Aliscore v2.0 [34]. Aliscore identifies sections in multiple sequence alignments which cannot be distinguished from random similarity. Gaps were treated as ambiguities and the maximum number of possible pairwise comparisons was analysed. Identified random similar sections were excluded with Alicutv2.2 (http://www.zfmk.de). Neighbor-Joining (NJ) trees based on K2P distances of COI and 28S rDNA were compiled in Mega v. 5.05 [29], and compared to check for congruence of retrieved clusters. The reliability of both trees was assessed with 5000 bootstrap replicates.

COI- and 28S rDNA-trees were edited in Corel Draw Graphics Suite X3 (www.coreldraw.softonic.de/).

To compare intraspecific and interspecific distances and to search for a DNA barcoding gap in Protura, a number of distance measures were analyzed with SpeciesIdentifier 1.7.8 [35].

To indirectly test for saturation, the ratio of transitions to transversions was evaluated using the software DAMBE [36] and visualized in a saturation plot.

## Results

### Primer-establishing for COI and 28S rDNA

The universal COI primer set LCO/ HCO [37] failed to yield any results under various PCR conditions. Iterative steps of primer refinement finally provided a set of six primer-combinations (Table 2). Due to high variation at both ends of the DNA barcoding fragment our primers are specific mostly at the genus level. These primers cover fragments from 660 to 872 bp length. Altogether, we were able to generate 89 COI barcodes with the unambiguously readable sequence-length ranging from around 480 to 860 bp.

**Table 2:** Primer-combinations for COI and 28S rDNA amplification.

Genus	Primer-Combination COI	Primer-Combination 28S rDNA
*Acerella*	CamF1 + DiplR1 (2)	D2a + D3b (2)
*Acerentomon*	ProtF2 + ProtR2 (37)	D1a + D3b (24, *A*. n. sp. gr. *doderoi*)
	LepF1 + DiplR1 (1, *A*. sp. gr. *microrhinus*)	D2a + D3b (16)
	CamF1 + DiplR1 (1, *A. italicum*) CamF1 + CamR1 (1)	
*Acerentulus*	CamF1 + DiplR1 (30)	D1a + D3b (18)
		D2a + D3b (4)
		D2aprot + D3b (3)
*Eosentomon*	LCO + ProtR2 (6)	D2aprot + D3b (3)
		D2a + D3b (2)
		D1a + D3b (1)
*Ionescuellum*	LepF1 + Prot R2 (8, *I. haybachae*)	D2a + D3b (14)
	CamF1 + DiplR1 (2, *I. carpaticum*) CamF1 + CamR1 (1)	
	LCO + ProtR2 (2, *I. silvaticum*)	
*Baculentulus*	LepF1 + DiplR1 (1)	–
*Filientomon*	LCO + ProtR2 (2)	D2a + D3b (1)
	CamF1 + CamR1 (1)	D1a + D3b (1)
		D2aprot + D3b (1)

Numbers of successfully sequenced specimens in parenthesis. Species names in parenthesis indicate that combinations seem to be species-specific.

Aside from COI, we tested a small set of 28S rDNA primers used by [30]. These primers work efficiently in PCR across almost the entire taxon sampling. To successfully sequence all specimens, the procedure of using a slightly derived forward, as well as internal primers proved necessary (Table 1). In total, we generated 82 sequences of 28S rDNA including the Divergent Domains and conserved regions 2 and 3, all of approximately 1000 bp length.

In COI for each genus the majority of representatives worked with the same primer-combination. Only a few specimens required a different primer combination, for example some representatives of *Acerentomon, Ionescuellum* and *Filientomon* (Table 2). COI primer combinations proved to be species- specific in *Ionescuellum*, as well as in *Acerentomon italicum* and *A.* sp. gr. *microrhinus* (Table 2).

The original primer set for the 28S rDNA fragment (D2a and D3b) worked in all representatives of *Acerella* and *Ionescuellum*. In all other genera, additional primer-combinations were required without any hint for species-specifity. In *Acerentulus*, *Eosentomon* and *Filientomon,* for example, all three possible combinations had to be applied to yield results in all representatives (Table 2).

### Species composition based on morphology

While determination of the specimens at the genus level preceded sequencing, species identification was performed after the molecular analyses by Julia Shrubovych, albeit without prior knowledge of the molecular results. Morphological species determination of the NDE vouchers of our three sampling sites resulted in 5 genera and 12 species (Table 3). Eight of the 12 species were sampled only at one site and therefore represent the range of variation within a single population.

**Table 3:** List of morphologically determined species of Protura investigated in this study.

Genus	Genus (Abbr.)	Species	Locality (Abbr.)
*Acerella*	*Aca*	*muscorum* (Ionsecu, 1930)	LB
*Acerentomon*	*Aco*	*italicum* Nosek, 1969	LB
		*maius* Berlese, 1908	TG
		sp. gr. *microrhinus* Tuxen	TG
		n. sp. gr. *doderoi*	LB
*Acerentulus*	*Ace*	*exiguus* Conde, 1944	LB, TG
		*tuxeni* Rusek, 1966	LB
*Eosentomon*	*Eos*	*cetium* Szeptycki & Christian 2000	LB, TG
		sp. gr. *delicatum*	LB
*Ionescuellum*	*Ion*	*carpaticum* (Ionescu, 1930)	LB, EK
		*haybachae* Nosek, 1967	LB, EK
		*silvaticum* Rusek, 1965	EK

Genus abbreviation: *Ace* = *Acerella*, *Aco* = *Acerentomon*, *Acu* = *Acerentulus, Eos* = *Eosentomon*, *Ion* = *Ionescuellum*; Locality abbreviation: LB = Leopoldsberg, TG = Twimberger Graben, EK = Eichkogel.

Soil samples from the Leopoldsberg yielded an *Acerentomon* species new to science belonging to the *doderoi* group (species description will follow in a separate paper). *Ionescuellum carpaticum* from Leopoldsberg and Eichkogel and *Acerentomon italicum* from Leopoldsberg represent first records for Austria.

While NDE itself never impeded determination, the preparation of whole-mounts in few instances caused wrinkle formation. Consequently a few specimens could be determined only to the genus level, and for some specimens determination at the species level was judged to be uncertain (abbreviation cf.). The investigated specimens include different developmental stages (larva II, maturus junior, preimago, and adult).

### Comparison between taxonomy and COI distances

**Figure 1:**
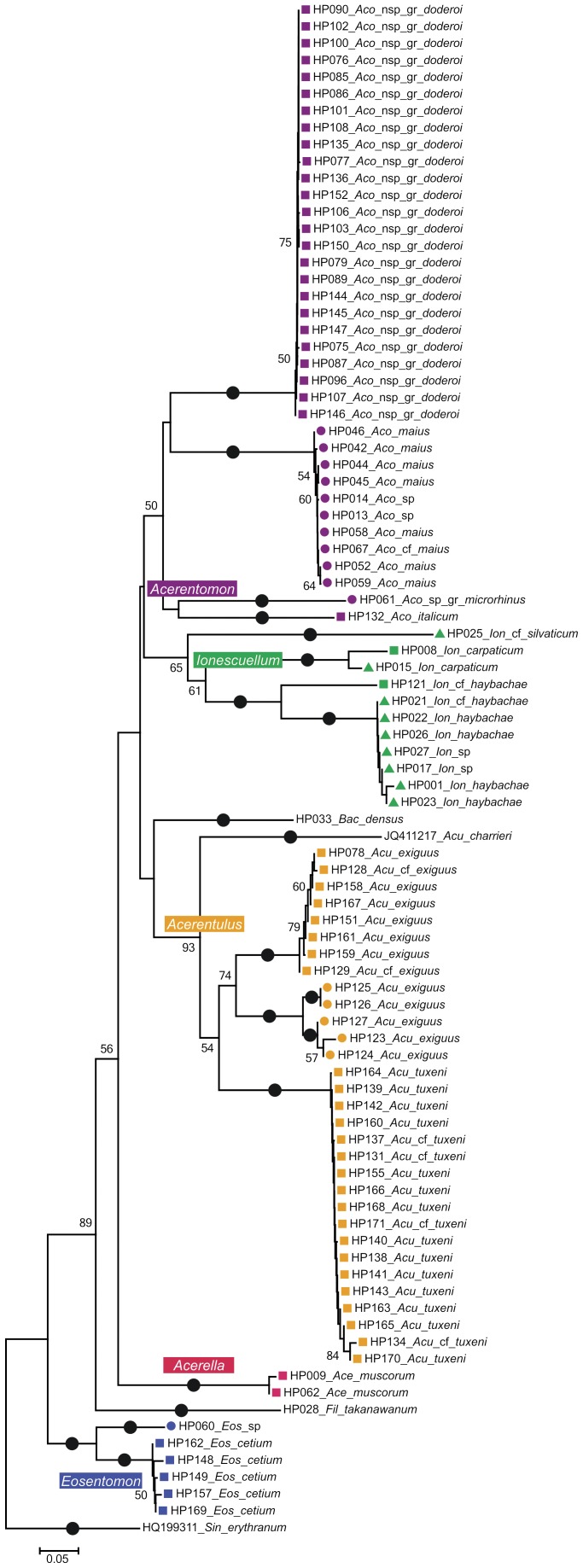
NJ tree based on K2P distances from 91 COI sequences of Protura. Newly sequenced specimens labeled with lab code number (HP), abbreviation for genus, and species name. Color code for genera: *Acerentomon* = violet, *Ionescuellum = *green, *Acerentulus* = orange, *Acerella* = red, *Eosentomon* = blue; Austrian sample sites are coded with different icons: Leopoldsberg = square, Eichkogel = triangle, and Twimberger Graben = circle. Bootstrap support (given below nodes) derived from 5000 replicates. Maximally supported clusters and subclusters are indicated by black dots. Genus abbreviations: *Aco* = *Acerentomon*, *Ion* = *Ionescuellum*, *Acu* = *Acerentulus*, *Ace* = *Acerella*, and *Eos* = *Eosentomon*.

The Neighbor-Joining (NJ) tree of the COI data contains 91 taxa with an alignment length of 657 bp, and shows 17 maximally supported subdivisions (Fig. 1). Remarkably, the analysis retrieved all identified genera in monophyletic clusters, and all morphologically determined species form monophyletic subclades. COI barcodes captured all species boundaries among the 12 species from Austria.

Within the *Acerentomon* cluster each subdivision reflects a species, each collected from a single locality. *Ionescuellum* is represented by three species. Two of them, *I. carpaticum* and *I. haybachae*, are reported from two different sites. Unfortunately, the only outlier of *I. haybachae* could not be determined unambiguously. The third major clade coincided with the genus *Acerentulus*. *A. exiguus* is the only species in which distances are high among individuals of the same population, but even there the values are still lower than distances between the two investigated populations. The only outlier within *Eosentomon* could not be determined to species level due to the low quality of the whole-mount.

Generally, Kimura-2-Parameter (K2P) distances within populations were very low, the only exception being *A. exiguus* from Twimberger Graben. Whenever two populations of the same species are covered, intraspecific distances are very high (up to 21.3% between populations of *I. haybachae*). Intrageneric distances are very high with maximal congeneric distances ranging around 30%, the highest distance value (44.7%) occurs between *I. silvaticum* and *I. haybachae* (Table 4).

**Table 4:** K2P distances in COI among species of Protura collected in Austria.

Species (Abbr.)	Max intra	Best intra	Max congen	Best match	Best inter
*Aca muscorum*	0	0	-	*Acu exiguus*	31.85–32.3
*Aco italicum*	-	-	33.3	*Aco* n. sp. gr. *doderoi*	31.2
*Aco maius*	0.3	0–0.3	33.3	*Aco* n. sp. gr. *doderoi*	28.0–28.5
*Aco* n. sp. gr. *doderoi*	0.6	0–0.2	33.0	*Aco maius*	28.0–28.8
*Aco* sp. gr. *microrhinus*	-	-	33.3	*Aco maius*	31.3
*Acu exiguus*	17.5	0–0.64	24.5	*Acu tuxeni*	20.5–23.6
*Acu tuxeni*	0.2	0–0.2	24.5	*Acu exiguus*	20.5–22.1
*Eos cetium*	1.8	0.31–0.84	15.6	*Eos* sp.	14.5–15.6
*Eos* sp.	-	-	25.6	*Eos cetium*	14.2
*Ion haybachae*	21.3	0–20.63	44.7	*Ion carpaticum*	33.3–34.5
*Ion carpaticum*	6.3	6.25	41.2	*Ion haybachae* & *Eos* sp.	33.6 & 32.3
*Ion silvaticum*	-	-	44.7	*Acu exiguus*	28.3

Species described by their maximal intraspecific (Max intra), and the range of the best intraspecific matches (Best intra), as well as the maximal congeneric interspecific distances (Max congen), the name of the best matching species (Best match), along with the range of their best interspecific distances (Best inter). In species, covered by a single representative, missing distances are marked by a short line. Species abbreviations as used in Tab. 3.

### Resolution power of COI and 28S rDNA

**Figure 2:**
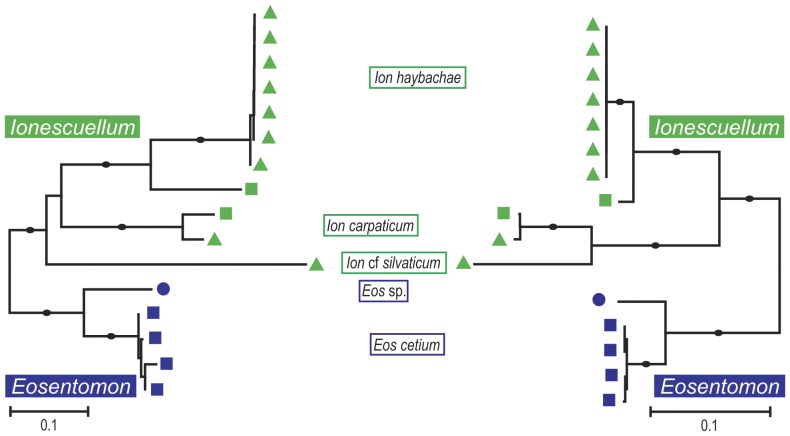
Comparison of COI and 28S rDNA in species discrimination of the genera *Ionescuellum* (*Ion*) and *Eosentomon* (*Eos*). NJ tree based on K2P distances of COI (left) and the mirrored 28S rDNA results (right). Bootstrap support (maximal support marked with full circles) derived from 5000 replicates is maximal for all species and polpulations. Color code for genera: *Acerentomon* = violet, *Ionescuellum* = green, *Acerentulus* = orange, *Acerella* = red, *Eosentomon* = blue; Austrian sample sites are coded with different icons: Leopoldsberg = square, Eichkogel = triangle, and Twimberger Graben = circle.

**Figure 3:**
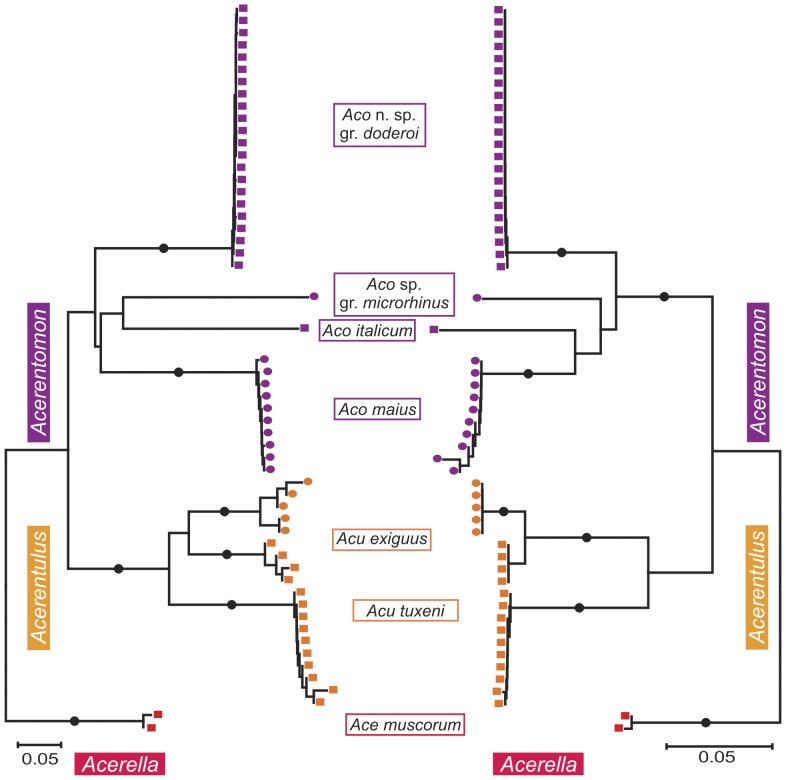
Comparison of COI and 28S rDNA in species discrimination of the genera *Acerentomon* (*Aco*), *Acerentulus* (*Acu*) and *Acerella* (*Ace*). Mirrored NJ tree based on K2P distances of COI (left) and 28S (right). Bootstrap support (maximal support marked with full circles) derived from 5000 replicates. Color code for genera: *Acerentomon* = violet, *Ionescuellum* = green, *Acerentulus* = orange, *Acerella* = red, *Eosentomon* = blue; Austrian sample sites are coded with different icons: Leopoldsberg = square, Eichkogel = triangle, and Twimberger Graben = circle.

The power of the sequenced 28S rDNA fragment to discriminate the studied specimens of Protura at the species level equals that of the COI barcoding fragment. Differences mainly pertain to branch length (Fig. 2 and 3).

Distances between populations are lower in 28S rDNA, albeit a proper comparison suffers from the necessity to exclude regions of highest variability since they are not unambiguously alignable among all investigated Protura. In *Ionescuellum haybachae* the maximum distance between populations from Eichkogel and Leopoldsberg are 3.1% (compared to 21.3% in COI), and between *Eosentomon cetium* from the Leopoldsberg and *E*. sp. from the Twimberger Graben 7.5% (15.6% in COI). Topological differences are low, but present within the genus *Ionescuellum* and profound in the placement of *Filientomon takanawanum*. In the NJ-tree based on 28S rDNA, *I. carpaticum* clusters with *I. silvaticum* (maximally supported), but with *I. haybachae* in the COI-tree (for complete NJ tree of 28S rDNA see Figure S1).

Distances of 28S rDNA sequences likewise are shorter within *Acerentomon*, *Acerentulus* and *Acerella* (see Tables S3, S4), as reflected in shorter internal branches in the tree. Important differences are evident in *Acerentulus exiguus* (see Table S2). Distances are very high in COI and the population of Twimberger Graben is further subdivided into two subclusters. In contrast 28S rDNA shows no distances within this population. The maximum distance between the populations of Twimberger Graben and Leopoldsberg is about seven times as high in COI (17.5%) as in 28S rDNA (2.5%). Some differences are apparent in the topology of the genus *Acerentomon*. In COI, a clade formed by *A*. sp. gr. *microrhinus* and *A. italicum* clusters with a clade containing *A. maius* and *A.* n.sp. gr. *doderoi*, while 28S rDNA retrieves *A*. n. sp. gr. *doderoi* as nearest neighbor to a clade comprising all remaining species of the genus and *Filientomon takanawanum* (see also Table S3). Bootstrap support for the respective nodes is low in both trees, and the respective internal branches are very short.

## Discussion

### Morphological taxonomy is accurately reflected in the molecular data

Morphological determination of Protura is extremely challenging and a skill that is restricted to a handful of taxonomists worldwide [4]. Identification at the species level strongly relies on subtle chaetotaxic characters, where the position and length ratio especially of certain foretarsal bristles play a crucial role [7], [38]–[39]. Both intraspecific variability and anomalies may further hamper identification [8], [40]–[42]. Furthermore it should be noted, that the sexual biology and mode of sperm transfer of Protura is still enigmatic [4] and we consequently have no possibility to check morphospecies from a biospecies concept perspective. Therefore, despite the great efforts of excellent taxonomists (see the critical catalogue of the Protura of the world by [39]), reliability of some morphospecies remains controversial; thus support from molecular data is desirable.

Despite suspected discrepancies, the clustering of our molecular data is exactly mirrored in the morphological determination. It should be mentioned that the species determination was performed without prior knowledge of the results from the molecular data. This clearly suggests that the morphospecies described by traditional taxonomy have a solid biological background and probably represent true biospecies. The seemingly marginal diagnostic characters used by traditional taxonomists obviously suffice for appropriate species identification of Protura. Our sequence data enables distinct species delimitation, which is supported by high interspecific distances. Furthermore, even different developmental stages and specimens, in which the quality of whole-mounts is not sufficient for species determination, can be appropriately allocated. Barcoding thus will foster further studies on morphological changes during postembryonic development.

### Extraordinarily high genetic distances within proturan barcodes

Our study revealed huge genetic distances among proturans, for both the maximal intraspecific (0–21.3%), as well as maximal congeneric interspecific distances (up to 44.7%) (Table 4, Tables S2, S3, S4, S5, and S6). Unique among published animal “record holders” of intraspecific variation, in Protura COI distances accurately allow to differentiate among all unambiguously determined species. This resolution power contrasts with most other groups, where saturation of COI sequences seems to prevent proper delimitation [43]–[46]. In Protura not only all morphospecies, but all 5 genera, as well as the three major lineages Sinentomata, Eosentomata, and Acerentomata [39], [47] were retrieved in the NJ-tree.

Testing our sequence data for sequence saturation, transversions are shown to be more common than transitions. Thus saturation is clearly present in COI sequences of Protura (Figure S1), like expected in ancient phylogenetic lineages, such as Protura. The retained resolution power may be due to (i) a low speciation rate within Protura, (ii) the low taxon sampling, or a combination of these effects. The high intraspecific distances among populations may indicate the presence of cryptic species. However, we refrain from premature species splitting, since the low dispersal capabilities may sufficiently explain the observed pattern.

### A closer look on the dispersal capability of Protura

Protura belong to the primarily wingless hexapods, which mostly show restricted dispersal capability compared to pterygote insects. Additional factors limiting proturan dispersal are their minute body size together with a slow mode of locomotion, as well as their euedaphic life style, which restricts them to deeper soil layers [48]–[49].

Passive dispersal by wind and water is known from other soil arthropods (Collembola: [50]–[51], Archaeognatha: [52], mites: [51]). Since Protura are strictly euedaphic, wind can be excluded as a possible dispersal medium. Floating in water is more conceivable, since many soil dwelling organisms are tolerant to hypoxia or anoxia [53], and pore space can be abruptly filled with water due to heavy rain or inundation. Several studies revealed that proturans can survive and show signs of active movement under water up to seven days [54], [Pomorski, personal comm.] and occur in soil habitats subjected to regular inundation [55]. Thus, if soil is washed out through heavy rain, specimens of Protura may be spread passively and thus be able to conquer new habitats.

### Misleading results when setting universal discrimination thresholds

Different operational criteria were proposed to permit species delimitation with molecular data including; (i) reciprocal monophyly [56], (ii) a barcoding gap [57]–[58], and (iii) absence of interlineage reproduction.

Reciprocal monophyly of proturan lineages is matched by the current data, since all species and genera cluster in monophyletic associations. [18] and [57] suggested interspecific distances 10x the average of intraspecific distances as a threshold for a barcoding gap. This would yield unrealistic to impossible values in Protura (up to 213%). Aside from this, our results show high intraspecific distances compared to moderate interspecific distances, as reported from other animals [45], [59]–[60]. Our highest maximal intraspecific distances (*Ionescuellum haybachae* 21.3%) exceeds smallest interspecific, congeneric distances (*Eosentomon* 14.5%) (Table 4). Such an overlap makes it difficult to set distance thresholds valid across the entire taxon sampling, and species delimitation solely dependent on DNA barcoding then becomes less effective (see also Tables S7 and S8). Furthermore, higher ranges of overlap must be expected once more closely related taxa are included. Thus, setting a general cut-off across clades becomes problematic and can lead to substantial errors in species identification, not only for Protura. For example, due to high sequence divergence among Protura it is not possible to decide unambiguously, whether the specimen of *Eosentomon*, in which the quality of the whole-mount did not allow for determination to species level, is conspecific with *Eosentomon cetium*. The observed distance may both represent intraspecific variation among distinct populations, or a species boundary. In the latter case, it would represent the lowest congeneric distance of COI within Protura.

Many authors explain high interspecific distances as an artifact of incomplete representation of the distributional range of a species (underestimating intraspecific distances), or as the failure to sample sister taxa (overestimating interspecific distances) [59], [61]. Given the limited taxon sampling of our study, both explanations have to be taken into consideration. In only four of the investigated species populations of different sampling sites are represented, and all of them are restricted to sample sites in Austria. All species, in which two populations are covered, revealed exceptionally high intraspecific distances. Therefore, we expect similarly high distances within other proturan species.

Searching for sister taxa will cause potential difficulties since phylogenetic relationships within the morphologically defined “species-groups” are usually unclear. One group of our taxon sampling may partially fulfill the demand of dense coverage among closely related species: It comprises the three species of the genus *Acerentomon* (*A. maius*, *A. italicum*, *A*. n. sp. gr. *doderoi*) which are representatives of the “*doderoi*-group”. On the one hand the maximal interspecific distance among these three species range from 31.9% to 33.3% and thus lies within the range of the maximal distance to *A*. sp. gr. *microrhinus*, which is the sole representative of the “*microrhinus*-group” (32.9% to 33.3%). On the other hand, the two species of *Acerentulus* have the lowest interspecific distance of our complete taxon sampling, although they represent different species-groups.

Beyond that, covering the entire geographical range of a species is often hampered especially in this hexapod group by the incomplete knowledge on its distribution. Research on proturan species distribution reveals huge gaps even within the relatively well investigated European areas (www.faunaeur.org). These gaps must be attributed to the underrepresentation of proturan research, in general, and particularly in broad ecological studies, but also to a lack of taxonomists working on Protura.

### Advantages of additional markers to support COI in challenging groups

The approach of DNA barcoding with only COI as standard marker and the chosen length of this fragment have been exhaustively debated (for reviews see [3], [62]).

To overcome these limitations we lengthened most sequences of COI by approximately 900 bp and used a fragment of 28S rRNA as a supplementary marker. The extension of the DNA barcoding fragment increases the phylogenetic signal and minimizes random variation in sequence divergence estimation. The use of an additional marker is highly recommendable since it represents an independent data set which allows for testing results obtained through DNA barcoding by COI [42]–[43], [63]–[64].

28S rRNA was chosen for several reasons. This gene is known to be built up of alternating highly conserved regions and variable Divergent Domains [65]. Therefore it provides conserved priming sites to design universal primers [65], as well as variability in primary sequence and length to delimitate between closely related taxa [66]. Furthermore, 28S rRNA as a nuclear gene has several advantages over an additional mitochondrial marker. The mitochondrial ribosomal DNA is generally assumed to evolve more rapidly than the nuclear genes [65]. This is of special importance for evolutionary ancient taxa, such as Protura, where saturation effects may become problematic. Finally, primers are well established in 28S rDNA, also for Protura. Despite the overall lack of public proturan sequences, much data is available regarding nuclear 18S rDNA and 28S rDNA [30], [67]–[69]. While 18S rDNA was previously used to resolve higher-level phylogenetic relationships within arthropods [65], [70], fragments of the 28S rDNA achieved more appropriate separation at the genus and species levels in Protura, Collembola and Diplura ([67] D3-D5, [69] D1-D11).

Our results demonstrate the high potential of the Divergent Domains 2 and 3 of 28S rDNA to accurately separate all investigated proturans to species and genus levels. As an advantage the presence of both conserved and variable regions leaves the 28S rDNA potentially informative not only for recent splits, but likewise for deeper nodes. Known length variation within the Divergent Domains [71], making sequence alignment a tricky and time consuming task, does not seem to impede the use of the fragment encompassing D2 and D3 in comparisons within Protura, but leaves comparisons of distance measures between the two genes problematic.

### Conclusions and future perspectives

Our study impressively shows that the DNA barcoding approach with the standardized COI marker region is applicable to accurately identify proturans at the species level. The high variation of COI at both primer sites demands the use of several primers to properly amplify the DNA barcoding fragment from all proturan species. One possibility to increase PCR, as well as sequencing success, would be the use of primer cocktails as implemented in [72].

Furthermore, our project revealed high intra- and interspecific distances within the taxon sampling. Due to this high variation of sequence divergence, an interspecific threshold is not yet applicable for Protura.

In our study we used an additional marker to investigate the molecular diversity of Protura, and both markers remarkably demonstrated the integrity of traditional morphology.

For future studies in Protura, we highly endorse the use of alternative markers, e.g. 28S rDNA, which are even more conserved and reliable for evolutionary ancient taxa. Especially in deep-rooted genetic lineages such as the primarily wingless hexapods more exhaustive taxon sampling will introduce problems of high genetic variation due to saturation of COI. Signals from additional markers, can provide independent support for species delimitation obtained through DNA barcoding. Otherwise, misinterpretation of results can lead to an overestimation of species richness or an underestimation of intraspecific variability. Additionally, restricted taxon sampling may lead to an underrepresentation of the complete genetic range of a species, as well as overlooking of sister species. We are aware that our taxon sampling is limited in terms of geographical distribution and the number of analyzed species. Nevertheless, we are confident that this pilot study will initialize a new avenue of research to improve and facilitate species delimitation and identification in Protura.

## Supporting Information

Figure S1 **Complete NJ tree based on K2P distances from 84 28S rDNA sequences (fragments D2-D3) of Protura.** Newly sequenced specimens labeled with lab code number (HP), abbreviation for genus, and species name. Color code for genera: *Acerentomon* = violet, *Ionescuellum* = green, *Acerentulus* = orange, *Acerella* = red, *Eosentomon* = blue; Austrian sample sites are coded with different icons: Leopoldsberg = square, Eichkogel = triangle, and Twimberger Graben = circle. Bootstrap support (given below nodes) derived from 5000 replicates. Genus abbreviations: *Aco* = *Acerentomon*, *Ion* = *Ionescuellum*, *Acu* = *Acerentulus*, *Ace* = *Acerella*, and *Eos* = *Eosentomon*.
(PDF)

Figure S2 **DAMBE substitution saturation plot for COI sequences of Protura.** The number of transitions (s) and transversions (v) is plotted against the K2P ( = K80) distance. The higher frequency of transversions compared to frequency of transitions clearly indicates saturation effects in our COI data set.
(PDF)

Table S1 **Species list of studied proturans, with Individual IDs, developmental stage, sampling location, used primer pair and Accession numbers given for each individual.**
(XLSX)

Table S2 **Maximal intraspecific K2P distances of COI and 28S rDNA sequences of investigated Protura.** Note that a proper comparison suffers from the necessity to exclude regions of highest variability with *Aliscore* since they are not unambiguously alignable among all investigated Protura. Calculated with SpeciesIdentifier 1.7.8.
(XLSX)

Table S3 **Maximal interspecific, congeneric K2P distances of COI and 28S rDNA sequences of investigated Protura.** Note that a proper comparison suffers from the necessity to exclude regions of highest variability with *Aliscore* since they are not unambiguously alignable among all investigated Protura. Calculated with SpeciesIdentifier 1.7.8.
(XLSX)

Table S4 **Smallest and mean interspecific, congeneric K2P distances of COI and 28S rDNA sequences in investigated Protura.** Note that a proper comparison suffers from the necessity to exclude regions of highest variability with *Aliscore* since they are not unambiguously alignable among all investigated Protura. Calculated with SpeciesIdentifier 1.7.8.
(XLSX)

Table S5 **Best match analysis of K2P distances of COI sequences of all investigated Protura.** Given are (i) the best intraspecific match, (ii) the best interspecific match, and (iii) information on the cluster of the best match. Calculated with SpeciesIdentifier 1.7.8.
(XLSX)

Table S6 **Best match analysis of K2P distances of 28S rDNA sequences of all investigated Protura.** Given are (i) the best intraspecific match, (ii) the best interspecific match, and (iii) information on the cluster of the best match. Calculated with SpeciesIdentifier 1.7.8.
(XLSX)

Table S7 **Cluster analysis of K2P distances of COI sequences of Protura.** All representatives of *Ionescuellum haybachae* can be found in a single cluster only at a value of 25%. At that time other species are already lumped into clusters containing multiple sequences. This illustrates that for COI no distance threshold can be given for species delimitation in Protura, which is valid across our entire taxon sampling. Calculated with SpeciesIdentifier 1.7.8.
(XLSX)

Table S8 **Cluster analysis Cluster analysis of K2P distances of 28S rDNA sequences of Protura.** All representatives of *Ionescuellum haybachae* can be found in a single cluster only at a value of 5%. At that time other species are already lumped into clusters containing multiple sequences. This illustrates that likewise for 28S rDNA sequences no distance threshold can be given for species delimitation, which is valid across our entire taxon sampling. Calculated with SpeciesIdentifier 1.7.8.
(XLSX)
