# Supplementary figures and images for: Where Taxonomy Based on Subtle Morphological Differences Is Perfectly Mirrored by Huge Genetic Distances: DNA Barcoding in Protura (Hexapoda)
Source: PLoS One. 2014 Mar 7;9(3):e90653. doi: 10.1371/journal.pone.0090653 (PMC3946556; doi:10.1371/journal.pone.0090653)

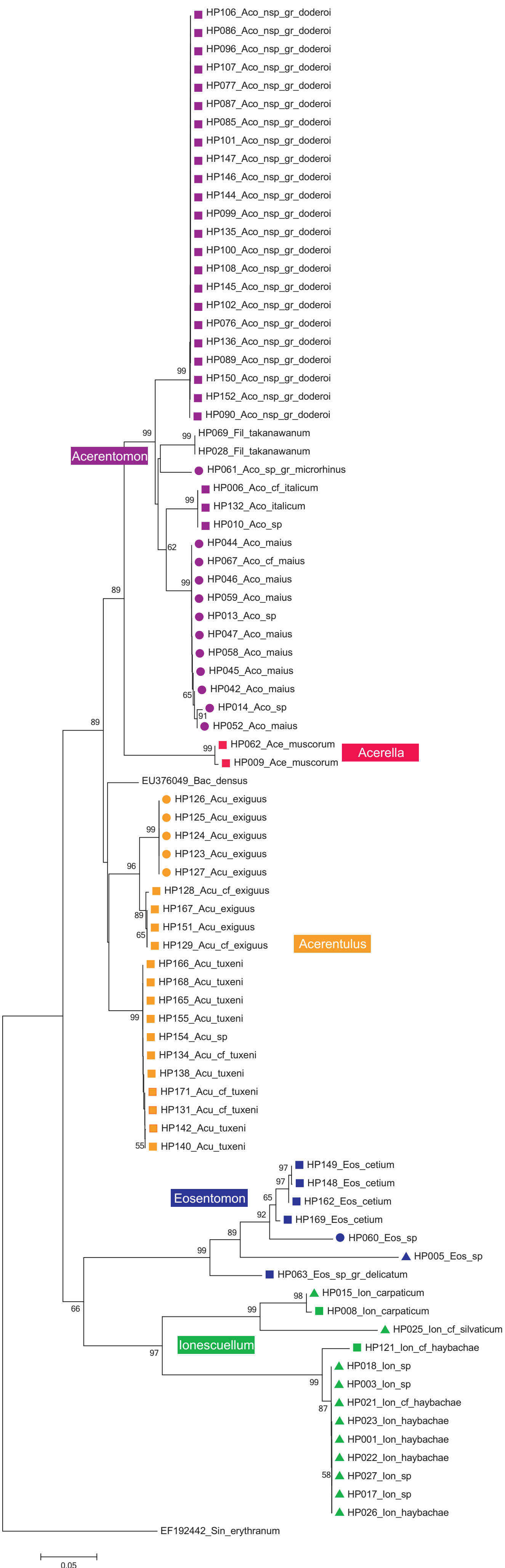

Supplement: Figure S1 — Complete NJ tree based on K2P distances from 84 28S rDNA sequences (fragments D2-D3) of Protura. Newly sequenced specimens labeled with lab code number (HP), abbreviation for genus, and species name. Color code for genera: Acerentomon = violet, Ionescuellum = green, Acerentulus = orange, Acerella = red, Eosentomon = blue; Austrian sample sites are coded with different icons: Leopoldsberg = square, Eichkogel = triangle, and Twimberger Graben = circle. Bootstrap support (given below nodes) derived from 5000 replicates. Genus abbreviations: Aco = Acerentomon, Ion = Ionescuellum, Acu = Acerentulus, Ace = Acerella, and Eos = Eosentomon. (PDF) [file pone.0090653.s001.pdf]

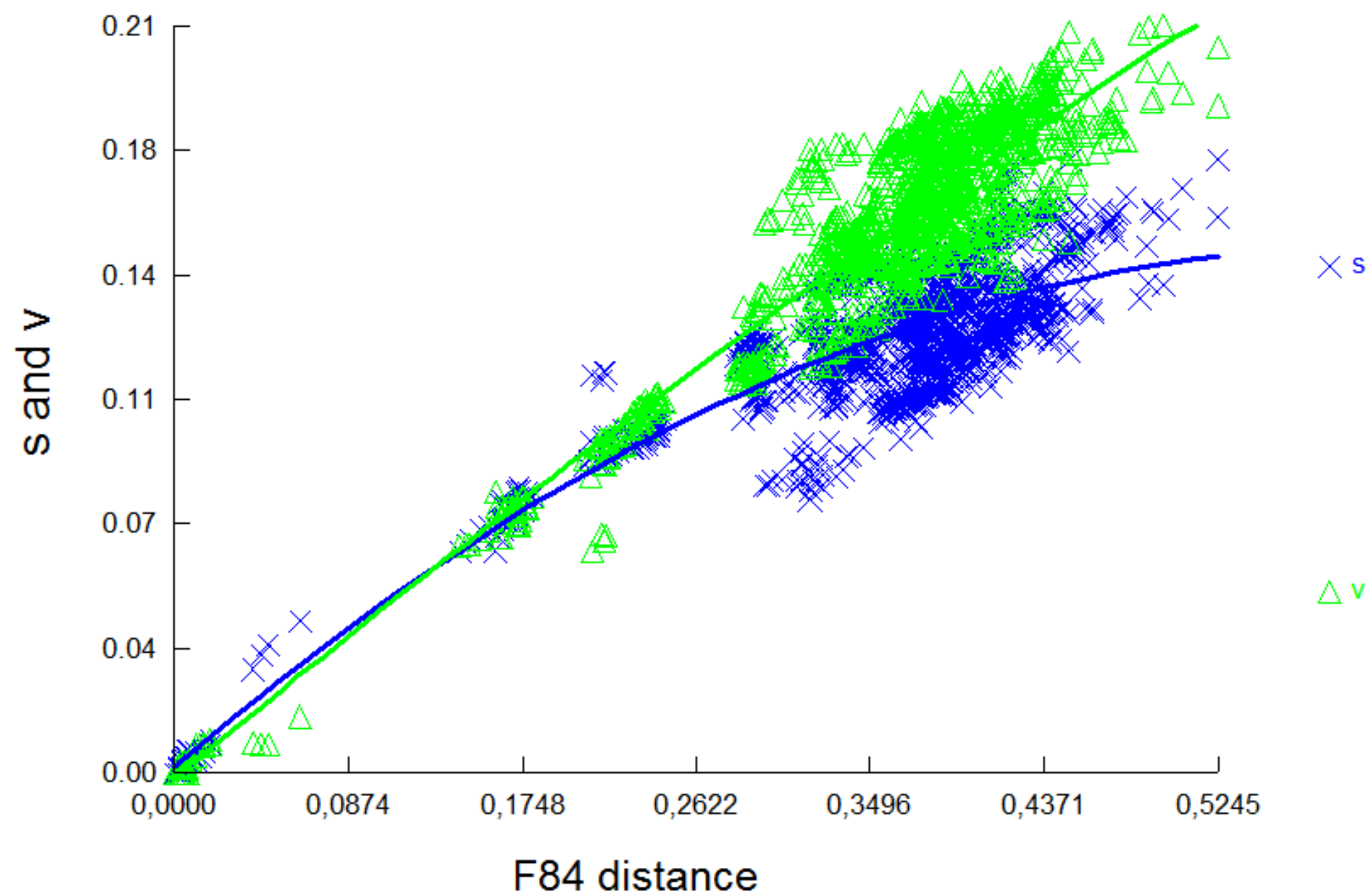

Supplement: Figure S2 — DAMBE substitution saturation plot for COI sequences of Protura. The number of transitions (s) and transversions (v) is plotted against the K2P ( = K80) distance. The higher frequency of transversions compared to frequency of transitions clearly indicates saturation effects in our COI data set. (PDF) [file pone.0090653.s002.pdf]
